# Closed-form design optimization for LLC converters with wide output voltage range based on FHA

**DOI:** 10.1038/s41598-025-32640-4

**Published:** 2026-01-10

**Authors:** Ahmed M. A. Hussein, Mostafa I. Marei, Mohammad H. Soliman

**Affiliations:** Electric Power and Machines Department, Ain Shams University, Cairo, 11535 Egypt

**Keywords:** LLC resonant DC–DC converter, Wide output voltage range, Parameter optimization, Improving efficiency, Optimal design, Energy science and technology, Engineering, Mathematics and computing

## Abstract

This paper presents a novel design optimization strategy for LLC resonant converters that enhances full-load efficiency while operating across wide input and output voltage ranges. Achieving regulation over a wide output voltage range imposes more stringent design constraints on the converter, demanding higher inductance ratio and wider switching frequency range compared to constant output voltage applications. While numerical optimization techniques are effective for determining the optimal parameters of the converter; however, this effectiveness comes at a substantial computational cost. This work establishes a set of closed-form analytical equations that not only constitute a complete, step-by-step procedure for optimal design without reliance on numerical solvers, but also provide a framework for analyzing design trade-offs. The proposed methodology distinguishes itself from conventional approaches by offering a systematic and non-iterative procedure that is computationally efficient for determining an optimal design. The proposed procedure is validated through the simulation of a 495 W LLC converter specified for a wide operational range with a 320–370 V input and a 35–165 V output. The converter achieved the full output voltage range at the worst-case conditions and attained peak efficiency near the full load, while maintaining soft switching across the entire operating range.

## Introduction

The LLC resonant converter has been widely used in DC–DC topologies for switched mode power supplies. In the literature, the LLC has been predominantly investigated in the context of constant output voltage applications^1–5^. Nevertheless, various authors have demonstrated that, by modulating the switching frequency, the LLC can manage wide-range output voltage regulation across large variations in both input voltage and load current^6–8^. Furthermore, the LLC can achieve robust soft switching under the worst-case conditions which is critical to minimizing electromagnetic interference (EMI) and maximizing efficiency. Switching the primary-side MOSFETs under zero-voltage switching (ZVS) eliminates reverse recovery losses, yielding lower total losses compared to zero-current switching (ZCS). Meanwhile, the rectifier diodes are switched under ZCS, further reducing losses^9^.

For constant output voltage applications, low inductance ratio and limited switching frequency range suffice; in contrast, wide output voltage applications necessitate simultaneously higher inductance ratio and broader frequency modulation, thereby further complicating the design optimization of the converter. Design procedures for employing the LLC as a variable voltage source with wide output voltage range have been proposed^10–13^. Furthermore, optimal design procedures for achieving the peak efficiency at a specified load for constant output voltage applications have been proposed^14,15^. Deriving closed form expressions for the component values is often unfeasible, given the high order nonlinear nature of the LLC circuit. Consequently, computer aided numerical optimization techniques have become prevalent for optimizing the converter^16^. There are several techniques commonly used to model the converter. The First Harmonic Approximation (FHA) method is widely employed to linearize the resonant network by retaining only its fundamental component, yielding an AC equivalent model, thereby significantly simplifying the analysis of the converter^17^.

Other approaches, like state-plane or time-domain analysis, are based on the exact modeling of the converter to provide precise description of the converter’s operation^18^. The exact models offer greater accuracy, nevertheless, at the expense of complicated equations which offer limited insights into the design tradeoffs and the influence of the converter parameters on the design constraints. In practice, the optimal design procedures employing the exact methods usually rely heavily on simulating the circuit with different component values^19,20^. Nevertheless, to avoid relying on simulators, methods based on numerical algorithms such as Stepwise Multi-Objective Parameter Optimization, and Surrogate Model have been proposed^21–23^. However, these methods require huge computational power and long execution time, thus limiting their utility.

In this paper, a novel optimal design procedure is introduced for the LLC resonant converter based on FHA. A step-by-step design procedure is proposed for optimizing this converter for wide output voltage range and improving efficiency at the full load while ensuring ZVS for the whole wide dynamic load range. The main advantage of the proposed methodology is that the converter design equations are derived analytically and expressed entirely in closed-form solutions, thus eliminating the need for the computationally expensive numerical methods used extensively in the predominant optimization methodologies proposed in literature. Furthermore, without requiring any auxiliary components to be added to the circuit, the proposed approach reduces cost, weight, and size while ensuring optimal performance.

In Sect. “LLC normalized DC output voltage”, the LLC resonant converter normalized dc output voltage based on the FHA approach is presented. The max and min voltage gain equations are stated in Sect. “Wide range operation necessary conditions”. An expression for approximating the conduction losses is presented in Sect. “Conduction losses approximation under the full-load condition”. The necessary equations for achieving wide-range operation and max efficiency while maintaining ZVS are derived in Sect. “The necessary conditions to achieve wide range and maximum efficiency simultaneously”. In Sect. ﻿“The proposed methodology derivation”, the equations governing the design procedure are derived. The design procedure independent parameters and the permissible parameter space are identified in Sect. “Identification of the independent parameters and the allowed parameter space”. The expressions for the dependent parameters in terms of the independent parameters are derived in Sect. “The converter dependent parameters in terms of the independent parameters”. In Sect. “The proposed updated design procedure”, the proposed design procedure and a step-by-step LLC converter parameters calculation are presented. The simulation results are discussed in Sect. “Simulation results”. Finally, the conclusions are listed in Sect. “Conclusion”.

## LLC normalized DC output voltage

**Fig. 1:**
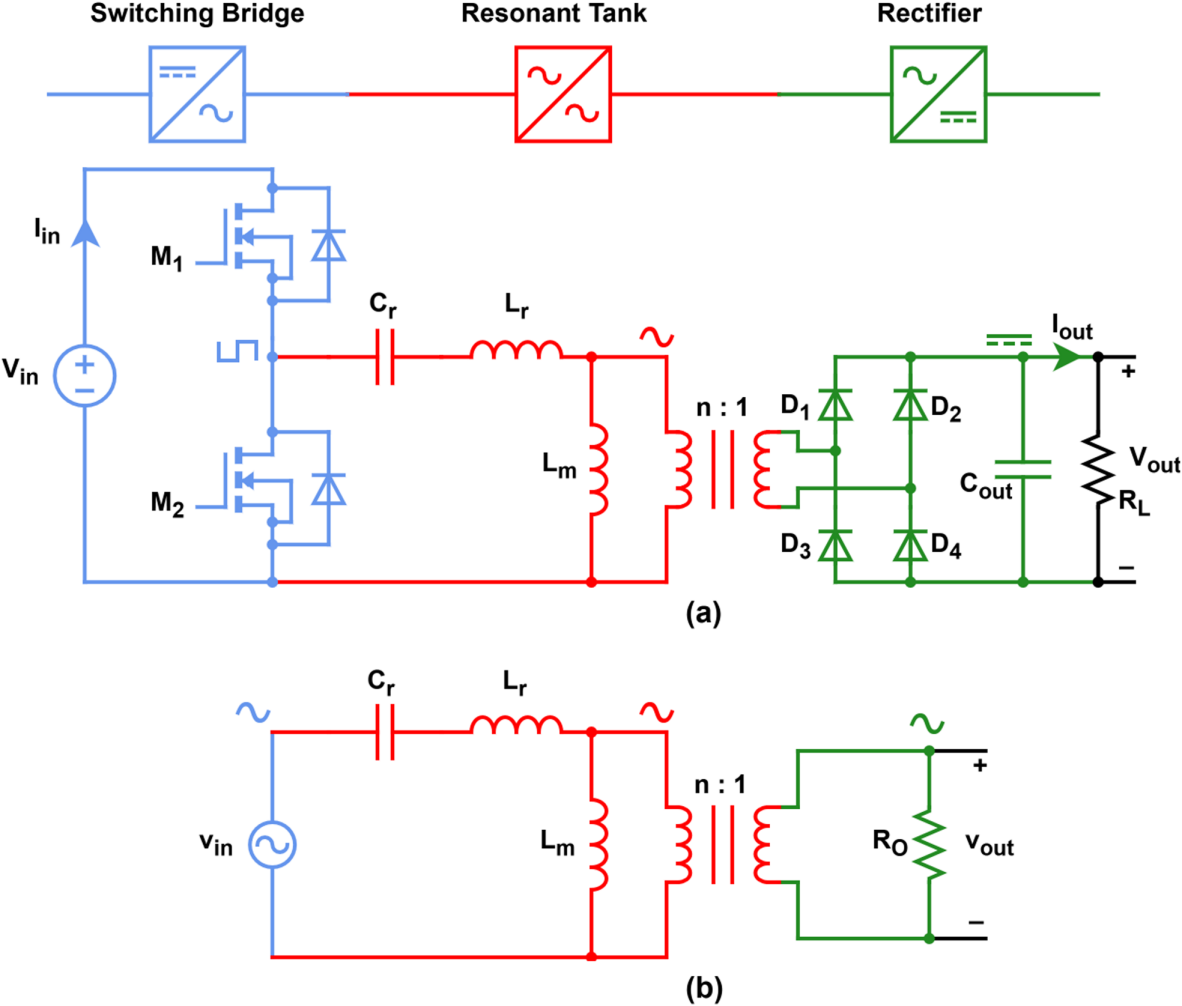
(**a**) LLC circuit topology, (**b**) LLC ac equivalent circuit.

The LLC resonant converter topology, depicted in Fig. 1, employs a half-bridge arrangement in which any imbalance in gate-drive timing or other sources of asymmetry produces a DC offset in the transformer’s primary current, risking core saturation. By inserting the resonant capacitor *C*_r_, this DC component is blocked, both preventing core saturation and forming the essential resonant network that governs the converter behavior. In this topology, the transformer’s turns ratio is *n* = *N*_*p*_/*N*_*s*_, where *N*_*p*_ and *N*_*s*_ denote the primary and secondary winding counts, respectively. The inductances *L*_*m*_ and *L*_*r*_ represent the magnetizing inductance and the primary-referred leakage inductance, respectively.

**Fig. 2:**
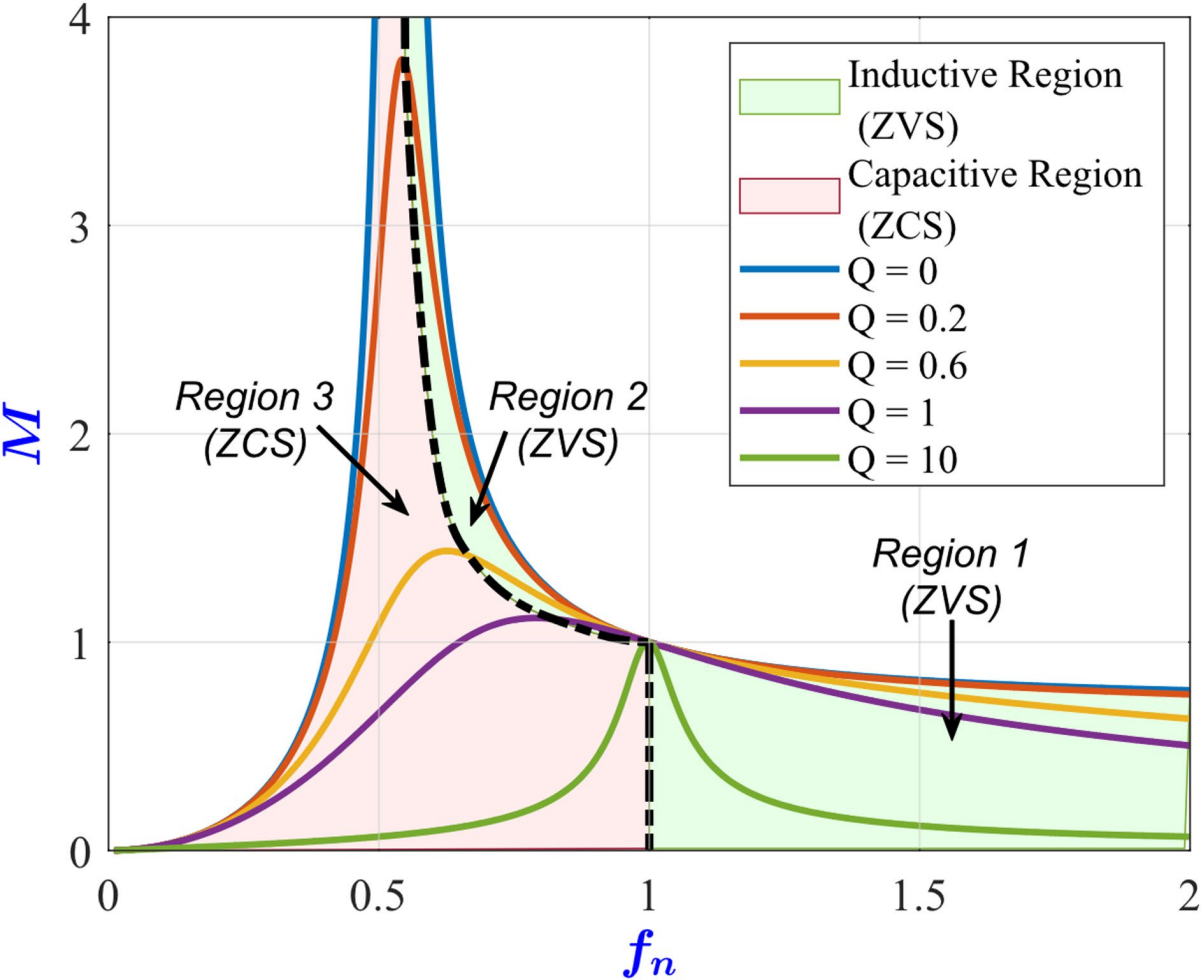
LLC voltage gain M versus normalized switching frequency f_n_ under different values of Q.

By employing the FHA method, the nonlinear circuit presented in Fig. 1a can be approximated by the linear circuit shown in Fig. 1b, where the combined effect of the rectifier and the load are represented by:

1 $$\:{R}_{o}=\frac{8}{{\pi\:}^{2}}{R}_{L}$$

where *R*_*L*_ is the load resistance^24^. Furthermore, the following parameters are also defined:

2 $$\:\lambda\:=\frac{{L}_{r}}{{L}_{m}}\:,\:{f}_{n}=\frac{{f}_{s}}{{f}_{r}}\:,\:Q=\frac{\sqrt{{L}_{r}/{C}_{r}}}{{n}^{2}{R}_{o}}\:,{f}_{r}=\frac{1}{2\pi\:\sqrt{{L}_{r}{C}_{r}}}\:,\text{}{f}_{\text{n,min}}=\:\frac{{f}_{\text{s,min}}}{{f}_{r}},{f}_{\text{n,max}}=\:\frac{{f}_{\text{s,max}}}{{f}_{r}}$$

where *λ* is the inductance ratio, *f*_*n*_ is the normalized switching frequency, *f*_*s*_ is the switching frequency, *f*_*r*_ is the resonant frequency, *Q* is the quality factor, *f*_*n,min*_ is the normalized minimum frequency, *f*_*s,min*_ is the minimum switching frequency, *f*_*n,max*_ is the normalized maximum frequency, and *f*_*s,max*_ is the maximum switching frequency. The voltage gain magnitude can be expressed^24^

3 $$\:M=\frac{2\:n\:{V}_{out}}{{V}_{in}}=\frac{1}{\sqrt{{(1+\lambda\:-\:\lambda\:/{{f}_{n}}^{2}\:)}^{2}+{\left(Q{f}_{n}\right(1-1/{{f}_{n}}^{2}\left)\right)}^{2}}}$$

where *V*_*out*_ is the output voltage, *V*_*in*_ is the input voltage, and *M* is the magnitude of the voltage gain of the converter. Figure 2 depicts the converter’s voltage gain characteristics (3) under various values of Q. The figure demonstrates the distinct operating regions of the converter. Zero-voltage switching (ZVS) is achievable wherever the gain curve slopes downward. Unlike a series resonant converter, which attains ZVS only above its resonant frequency, the LLC configuration can achieve ZVS both below and above resonant frequency. However, operating above the resonant frequency exhibits the same drawbacks as the series converter, such as requiring a wide frequency range for voltage regulation. The optimal operating frequency is at resonance, where switching losses and circulating currents are minimal. In practice, variations in the input voltage and load typically necessitate adjusting the operating frequency above or below the resonant frequency.

The following sections will present the proposed methodology for LLC converter design. Table 1 summarizes the comparison between the proposed methodology and the other methodologies in the literature.

**Table 1:** Qualitative comparison between different optimal design methodologies.

Comparison point	Proposed	^10^	^15^	^19^	^21–23^	^25^	^26^	^27^
Supports wide range output voltage operation	Yes	Yes	No	Yes	Yes	No	Yes	Yes
Supports wide range input voltage operation	Yes	Yes	Yes	No	Yes	Yes	Yes	No
Optimizes the efficiency	Yes	No	Yes	Yes	Yes	Yes	Yes	Yes
Requires multiple iterations	No	Yes	No	Yes	Yes	Yes	No	No
Based on numerical optimization	No	No	No	No	Yes	No	No	No
Based on closed form expressions	Yes	Yes	Yes	Yes	No	Yes	Yes	Yes
Based on FHA/TDA (time domain analysis)	FHA	FHA	FHA	TDA	TDA	TDA	TDA	FHA
Computation time required	Low	Low	Low	Mid	High	Mid	Mid	Low
Design methodology ease of use	Easy	Mid	Easy	Mid	Hard	Mid	Mid	Easy

Based on the comparison presented in Table 1, the proposed method demonstrates several key advantages over existing approaches:

- Supporting wide voltage range operation for both input and output^15,19,25,27^.
- Avoiding the need for multiple iterations^10^ and for solving a large set of equations numerically^26^.
- Eliminating reliance on computationally intensive numerical optimization^21–23^.

The main limitation of the proposed approach compared to other published methods is its reliance on FHA and not the more accurate TDA.

## Wide range operation necessary conditions

**Fig. 3:**
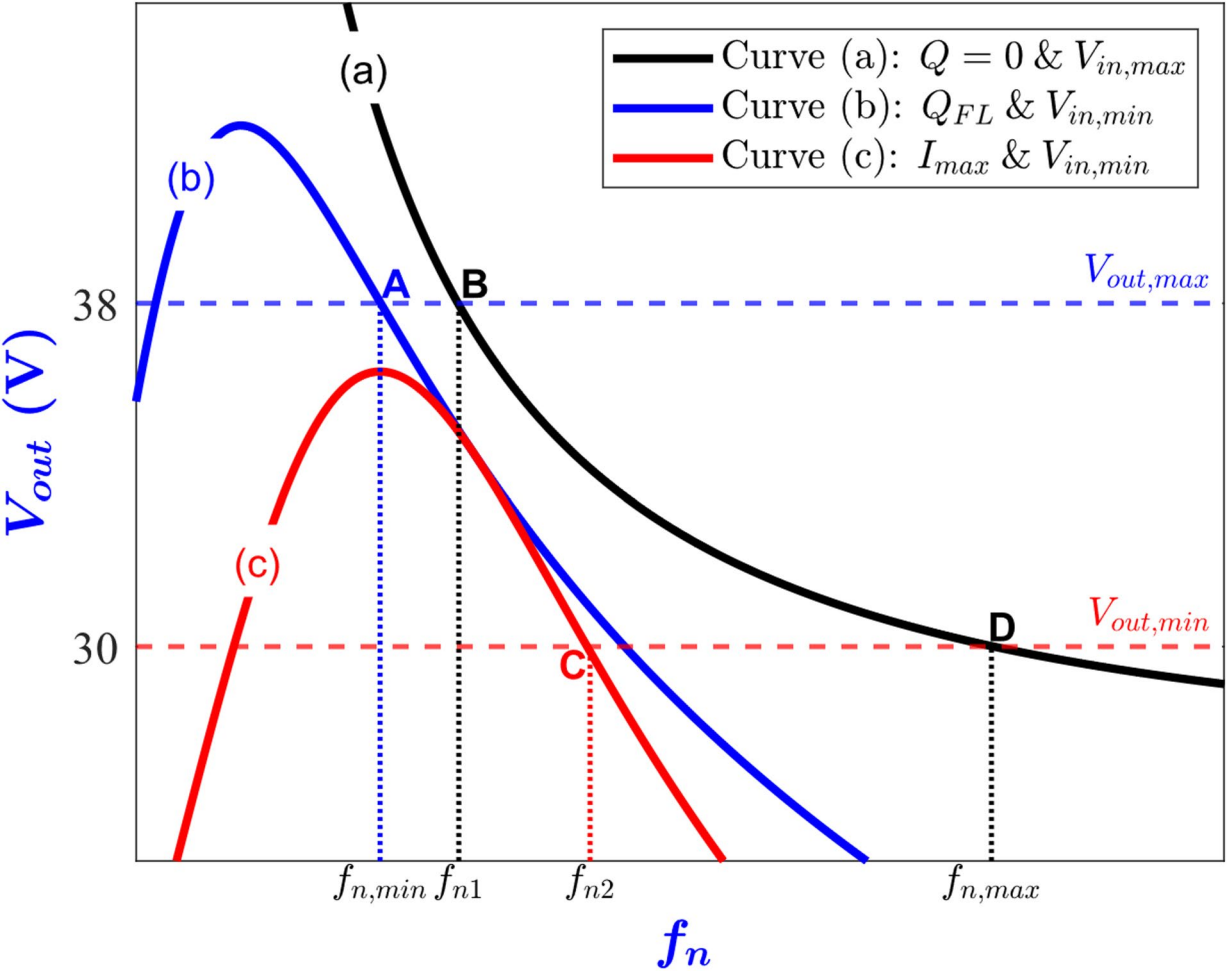
LLC output voltage V_out_ versus normalized switching frequency f_n_ under different conditions. (**a**) V_in_ = 65 V, Q = 9 × 10^−3^ . (**b**) V_in_ = 60 V, Q = 0.755. (**c**) V_in_ = 60 V, Q = 1.27.

In this section, the necessary conditions for achieving the wide range operation are derived. From (3), the relationship between the dc output voltage of the converter and the normalized switching frequency *f*_*n*_ has been depicted in Fig. 3 for various values of the quality factor (Q) and different input and output voltage conditions^7^. In this figure, curve (a) represents the converter operating under no-load with the input voltage at its maximum. However, curve (b) corresponds to the scenario where the converter is supplied with the minimum input voltage under the full-load condition. While curve (c) depicts the scenario of minimum input voltage together with maximum load current. Accordingly, segment A-B is the switching frequency range for regulating the output voltage at its maximum value, while the C-D interval is for regulating the output voltage at its minimum value.

More specifically, point A represents operating at the minimum *V*_*in*_ and the maximum *V*_*out*_ at the full load condition, while point B represents operating at the maximum *V*_*in*_ and the maximum *V*_*out*_ at no load. Moreover, point C represents operating at the minimum *V*_*in*_ and the minimum *V*_*out*_ while drawing the max load current. Finally, point D represents operating at the maximum *V*_*in*_ and the minimum *V*_*out*_ at no load. From Fig. 3, the operating points of the converter for all variations in *V*_*in*_, *V*_*out*_ and load current are located between points A and D. Consequently, achieving points A and D automatically guarantees achieving the other points over the entire operating range of the converter. Thus, the necessary constraints for the wide range operation are achieving the maximum voltage gain (*M*_*max*_) at point A and the minimum voltage gain (*M*_*min*_) at point D.

The maximum voltage gain occurs when the minimum input voltage *V*_*in, min*_ is applied to the converter and the maximum output voltage *V*_*out, max*_ is requested under the full-load condition. From (3), the maximum voltage gain can be expressed as

4 $$\:{M}_{max}=\:\frac{2\:n\:{V}_{out,max}}{{V}_{in,min}}\:=\frac{1}{\sqrt{{(1+\lambda\:-\:\lambda\:/{{f}_{n,min}}^{2}\:)}^{2}+{\left({Q}_{FL}\right(1-1/{{f}_{n,min}}^{2}\left)\right)}^{2}}}$$

The permissible input voltage range for realizing the maximum output voltage is determined by the converter’s peak voltage gain. Consequently, the converter’s gain curve must exhibit a sufficiently high peak to accommodate the full range of expected input voltages. Furthermore, the ZVS is lost when operating at a frequency below that of the peak gain point^19^, the capacitive region as illustrated in Fig. 2. To ensure robust ZVS operation during transient conditions, a safety margin is necessary when specifying the maximum gain. In practice, it is common to allocate a 10–20% safety margin above the required maximum gain (4), thus guarantying reliable ZVS. Additionally, under light-load or no-load conditions (where *Q* approaches zero), the converter must be capable of achieving the minimum output voltage *V*_*out, min*_ even when the maximum input voltage *V*_*in, max*_ is applied. From (3), the minimum voltage gain can be expressed as

5 $$\:{M}_{min}=\:\frac{2\:n\:{V}_{out,min}}{{V}_{in,max}}\:=1/(1+\lambda\:-\lambda\:/{{f}_{n,max}}^{2})$$

## Conduction losses approximation under the full-load condition

**Fig. 4:**
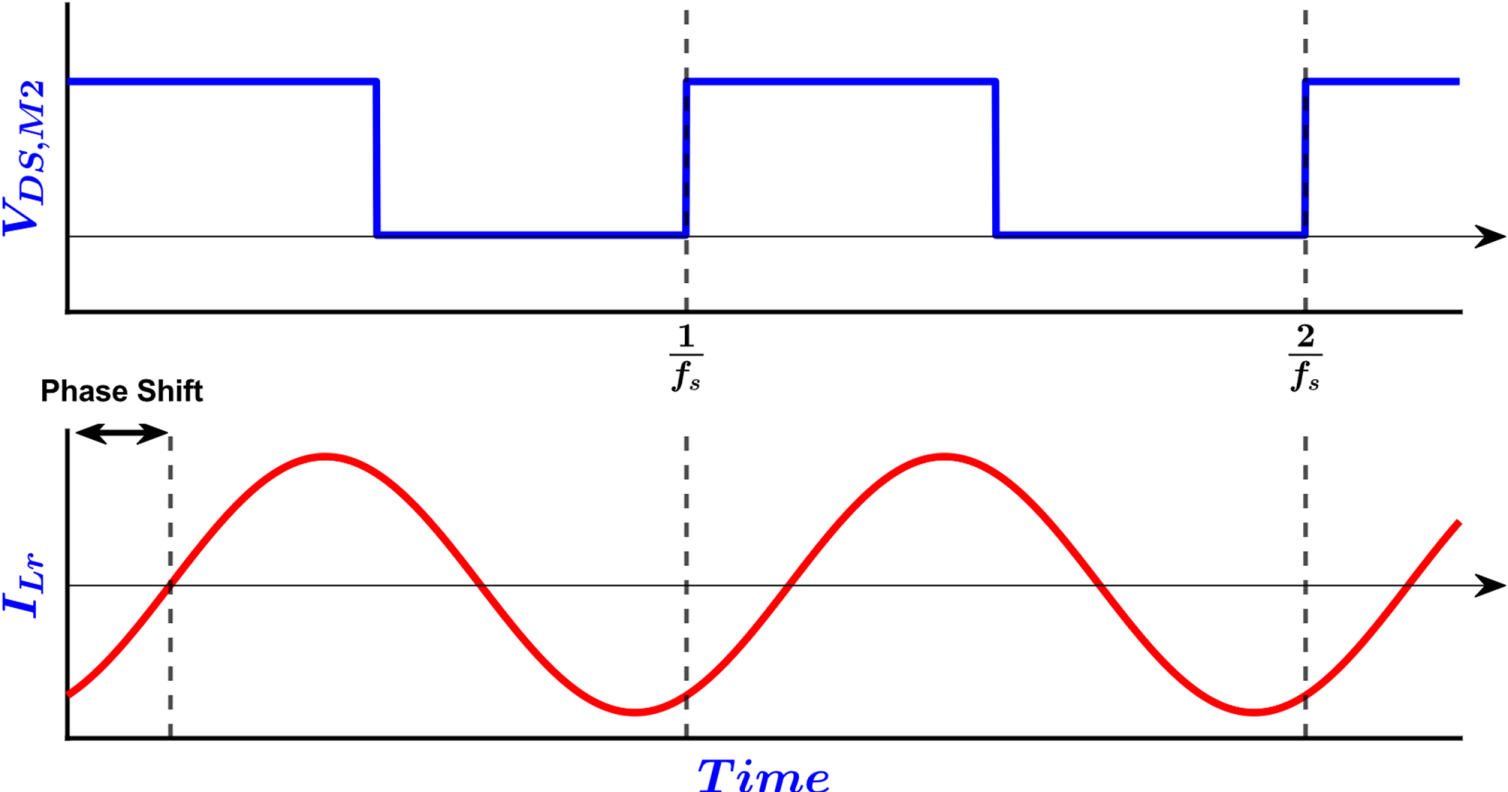
LLC waveforms, low side MOSFET drain source voltage V_DS, M2_, and resonant inductor current I_Lr_ (MOSFET current).

To ensure high efficiency, the converter must be designed in such a way as to achieve max efficiency near the full-load operating point, since this is the most probable operating point over the converter’s lifetime. To estimate the converter’s efficiency, an approximate expression for the conduction losses of the converter was derived^10^. The approximate expression is based on the following assumptions:

- The current drawn by the MOSFETs during the on-state is approximated with its first harmonic as shown in Fig. 4.
- The overall resistance seen by that current is lumped into a single equivalent resistance.
- The average power dissipation is evaluated by integrating the product of this equivalent resistance and the square of the approximated sinusoidal current over one switching period.
- The maximum value for the conduction losses is realized at Point A in Fig. 3 (more specifically at *V*_*out, max*_, *V*_*in, min*_, *f*_*n, min*_, and *Q*_*FL*_).

The approximate expression for the normalized peak conduction losses

6 $$\:{P}_{loss}=\frac{{P}_{cond,max}}{{r}_{DS}{V}_{in,norm}^{2}\:\left({L}_{r}/{C}_{r}\right)}=\frac{1}{{\pi\:}^{2}}\frac{{({\lambda\:}^{2}+{Q}_{FL\:}^{2}{f}_{n,min}^{2})}^{2}}{{Q}_{FL\:}^{2}{f}_{n,min}^{4}}{(V}_{in,min}^{2}/{V}_{in,norm}^{2})\:\:$$

Where $$\:{V}_{in,norm}=\:({V}_{in,min}+{V}_{in,max})/2$$.

## The necessary conditions to achieve wide range and maximum efficiency simultaneously

In this section, the necessary conditions that must be satisfied by the converter to achieve output voltage wide range operation, attain max efficiency near the full-load condition, and ensure ZVS across the entire operating range are derived.

### Conditions for operating in the inductive region

Since $\:0\le\:Q\le\:{Q}_{FL}$ for all permissible values of *Q*, where *Q* achieves 0 at no-load and *Q*_*FL*_ at full-load. From^24^, a sufficient condition for the converter to be operating inside the ZVS region for all permissible *Q*,

7 $$\:{Q}_{FL}\le\:\sqrt{\frac{\lambda\:}{1-{f}_{n,min}^{2}}-\frac{{\lambda\:}^{2}}{{f}_{n,min}^{2}}}$$

Where equality holds when operating at the edge of the ZVS region since the boundary between the ZVS and ZCS region is part of the ZVS region. To ensure that (7) yields non-negative real value for *Q*_*FL*_, the following conditions must be satisfied

8 $$\:0<\lambda\:\le\:\frac{{f}_{n,min}^{2}}{1-{f}_{n,min}^{2}}$$

9 $$\:0<{f}_{n,min}<1\:$$

### Achieving maximum efficiency at the full-load condition

To place the peak of the converter’s efficiency at the full-load operating point (point A in Fig. 3), the efficiency can be expressed at any load in terms of *Q* as

10 $$\:\eta\:\left(Q\right)=\frac{{P}_{out}\left(Q\right)}{{P}_{out}\left(Q\right)+{P}_{loss}\left(Q\right)}$$

Assuming that the output power is constant and equal to the full load power (10), can be rewritten as

11 $$\:\eta\:\left({Q}_{FL}\right)=\frac{{P}_{out,FL}}{{P}_{out,FL}+{P}_{loss}\left({Q}_{FL}\right)}$$

Maximizing the efficiency becomes equivalent to minimizing the power losses

12 $$\:{max}\left[\eta\:\right({Q}_{{FL}}\left)\right]\equiv\:{min}\left[{P}_{loss}\right({Q}_{FL}\left)\right]\equiv\:\:\:\:\frac{\partial\:{P}_{loss}\left({Q}_{FL},{f}_{n,min},\lambda\:\right)}{\partial\:{Q}_{FL}}=0$$

Differentiating (6) with respect to *Q*_*FL*_ results in,

13 $$\:{Q }_{FL}=\frac{\lambda\:}{{f}_{n,min}}$$

Differentiating (6) with respect to *Q*_*FL*_ twice and applying (13) then simplifying

14 $$\:\frac{{{\partial\:}^{2}P}_{loss}\left({Q}_{FL},{f}_{n,min},\lambda\:\right)}{{\partial\:{Q}_{FL}}^{2}}=\:\frac{8\:{(V}_{in,min}^{2}/{V}_{in,norm}^{2})\:\:}{{\pi\:}^{2}}>0$$

The second derivative test in (14) proves that (13) minimizes the power loss, thus maximizing the efficiency.

**Fig. 5:**
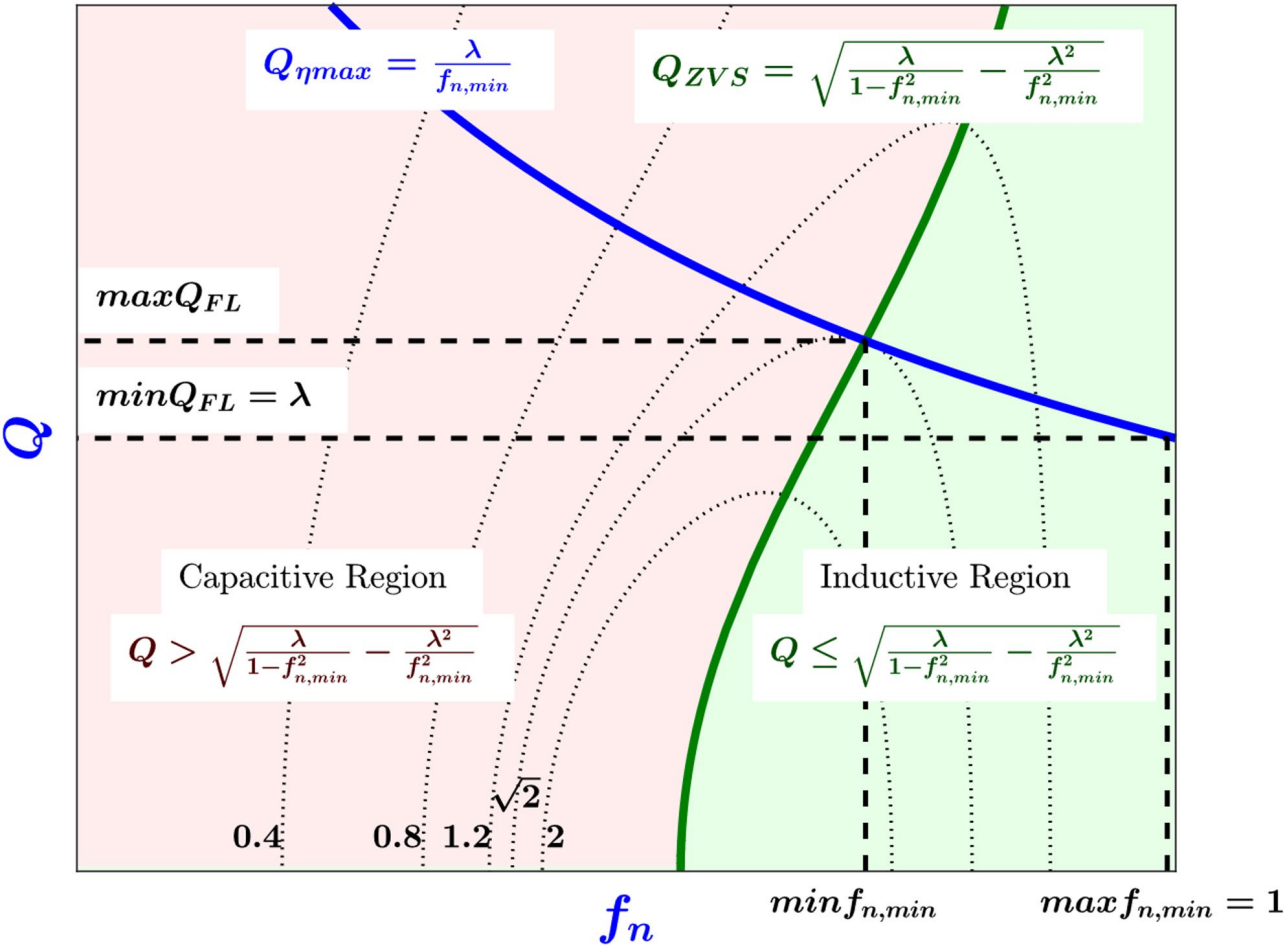
Contour map of the voltage gain M in terms of f_n, min_ and Q at a specific λ, the capacitive region is in pink while the inductive region is in green, Q_ZVS_ is the equation for the Q at the edge of ZVS (dark green), Q_ηmax_ is the equation for the Q that maximizes the efficiency (blue).

To visualize (13), Fig. 5 plots the contour maps of the voltage gain *M* at a specific *λ* with *f*_*n, min*_ on the x-axis and *Q* on the y-axis. The graph is split into two regions, the inductive region where the ZVS condition (7) is satisfied, while the capacitive region where the ZVS condition is not satisfied (ZCS is satisfied instead). The *Q*_*ZVS*_ curve separates the two regions, and it is part of the inductive region, according to (7). The *Q*_*ηmax*_ curve is the locus of all points where the efficiency is maximum (13). From this figure, to maximize the efficiency at the full load condition and achieve ZVS, the converter must be designed such that point A, in Fig. 3, is placed at the intersection of the *Q*_*ηmax*_ curve and the inductive region as shown in Fig. 5.

## The proposed methodology derivation

### Identification of the independent parameters and the allowed parameter space

Substituting the max efficiency *Q*_*FL*_ from (13) into ZVS region inequality (7)

15 $$\:\frac{\lambda\:}{{f}_{n,min}}\le\:\sqrt{\frac{\lambda\:}{1-{f}_{n,min}^{2}}-\frac{{\lambda\:}^{2}}{{f}_{n,min}^{2}}}$$

Simplifying (15), gives the following inequalities

16 $$\:0<\lambda\:\le\:\frac{{f}_{n,min}^{2}}{2-2{f}_{n,min}^{2}}$$

17 $$\:0<{f}_{n,min}<1\:\:$$

Equation (15) is equivalent to (16) if and only if (17) is satisfied. To ensure that the derivation is mathematically sound, the inequalities (16 and 17) must satisfy the validity conditions (8, 9). To prove this, it can be observed that (9 and 17) are identical, and through some straightforward algebraic manipulations it can be shown that the region defined by (16 and 17) is situated entirely within the boundaries of the region defined by (8). Therefore (16 and 17), satisfy (8 and 9).

**Fig. 6:**
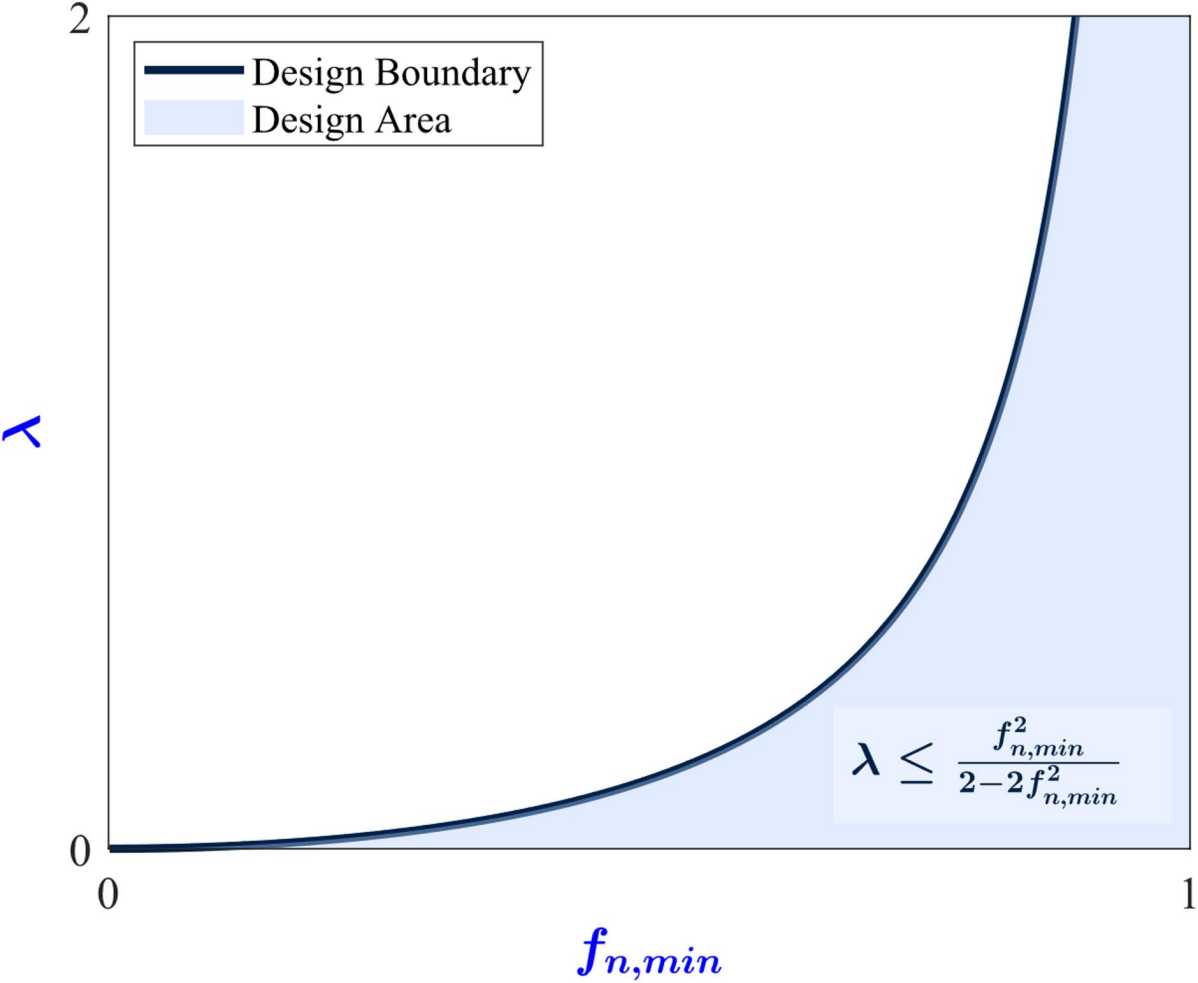
The design area (permissible region in f_n, min_ and λ parameter space), where ZVS and max efficiency at full load are achieved.

Equations (16 and 17) are necessary conditions to ensure achieving wide range operation, while maintaining ZVS across the whole operation, and attaining the max efficiency at the full load. Therefore, to ensure that the converter achieves these conditions, *f*_*n, min*_ and *λ* must be chosen in such a way as to satisfy the conditions (16 and 17). This result is visualized in Fig. 6. Within the parameter space spanning all combinations of *f*_*n, min*_ and *λ* representing the different possible designs of the converter, (16) and (17) establish a region of permissible values, effectively restricting the permissible parameter combinations that can be used to design the converter (the design area).

This can be interpreted as follows, the objectives of achieving wide range operation, maintaining ZVS for the entire operating range, and attaining max efficiency at the full load effectively restricting all of the converter’s degrees of freedom except for two. In other words, *f*_*n, min*_ and *λ* are the only independent parameters of the converter while the rest of the converter’s parameters can be expressed in terms of *f*_*n, min*_ and *λ*. Although *f*_*n, min*_ and *λ* are independent design parameters, their permissible values are not entirely independent. In fact, a dependency exists between the two where the selection of one parameter’s value restricts the feasible range of the other as can be seen in Fig. 6. The next step in the design procedure is to express each parameter of the converter in terms of *f*_*n, min*_ and *λ*.

### The converter dependent parameters in terms of the independent parameters

#### The maximum voltage gain

To express the max voltage gain *M*_*max*_ in terms of *f*_*n, min*_ and *λ*, substituting in the voltage gain Eq. (4) using the *Q*_*FL*_ at max efficiency (13),

18 $$\:{M}_{max}=\frac{{f}_{n,min}^{2}}{\sqrt{{f}_{n,min}^{4}+2{f}_{n,min}^{2}(-1+{f}_{n,min}^{2})\lambda\:+2{(-1+{f}_{n,min}^{2})}^{2}{\lambda\:}^{2}}}$$

It can be shown that for the entire range defined by (17) for *f*_*n, min*_ and (16) for *λ* (18), is positive real. Therefore, there are no additional validity conditions. Moreover, combining the constraints of *f*_*n, min*_ (17) and *λ* (16) with (18), then simplifying gives:

19 $$\:1<{M}_{max}\le\:\sqrt{2}$$

This is the range of the permissible values for *M*_*max*_. The conditions under which the lower and upper bounds are attained are derived next. The Lower bound in (19) is achieved when the voltage gain of the converter approaches unity which happens as *f*_*n, min*_ approaches 1 regardless of the value of *λ*. The upper bound in (19) is achieved when the gain is maximized while remaining in the ZVS region, this happens along the edge of the ZVS region. To prove this, substituting the expression for the *Q*_*FL*_ at max efficiency (13) into the expression for the *Q*_*FL*_ at the edge of the ZVS region (7) when the equality holds), then simplifying gives

20 $$\:\lambda\:=\frac{{f}_{n,min}^{2}}{2-2{f}_{n,min}^{2}}$$

21 $$\:0<{f}_{n,min}<1\:\:$$

Deriving Eq. (19) again but this time using (20) instead of (16) results in

22 $$\:{M}_{max}=\sqrt{2}$$

Equation (22) shows that the upper bound in (19) is attained when the converter maximum voltage gain point is placed at the edge of the ZVS region while also achieving maximum efficiency at full load. In other words, when point A in Fig. 3 is placed at the intersection of the *Q*_*ηmax*_ curve and the *Q*_*ZVS*_ curve in Fig. 5.

Accordingly, to increase the maximum voltage gain of the converter *M*_*max*_, the converter maximum voltage gain point (point A in Fig. 3) must be placed closer to the edge of the ZVS region (the Q_ZVS_ curve in Fig. 5). To achieve this, *λ* must be chosen in (16) to be closer to its upper bound. In summary, the designer can increase the maximum voltage gain of the converter by choosing *f*_*n, min*_ and *λ* closer to the left boundary of the allowed region in Fig. 6.

#### The transformer’s turns ratio

To express the transformer’s turns ratio n in terms of *f*_*n, min*_ and *λ*, solving (4) for *n*,

23 $$\:n=\:\frac{{M}_{max}\:{V}_{in,min}}{2\:\:{V}_{out,max}}$$

To express the minimum voltage gain *M*_*min*_ in terms of *f*_*n, min*_ and *λ*, substituting (23) into (5),

24 $$\:{M}_{min}={M}_{max}\:\frac{{V}_{in,min}{\:V}_{out,min}}{{V}_{in,max}{\:V}_{out,max}}\:=\:{M}_{max}\:\alpha\:$$

25 $$\:\alpha\:=\frac{{V}_{in,min}{\:V}_{out,min}}{{V}_{in,max}{\:V}_{out,max}}=\frac{{V}_{in,min}}{{V}_{in,max}}\times\:\frac{{\:V}_{out,min}}{{\:V}_{out,max}}$$

The expression for *M*_*min*_ in (24) can be written in terms of *f*_*n, min*_ and *λ* by substituting for *M*_*max*_ using (18). The ratio of voltages in (24) will play an important role in the design procedure. Therefore, it is introduced in (25) as the “voltage ratio product” and given the symbol “*α*”. The voltage ratio product *α* is defined as the product of two voltage ratios, the ratio of the input voltage range and the ratio of the output voltage range. If the converter is acting as a constant voltage source and operates only at a specific input voltage, then *α* will equal one. However, if the converter is operating as a wide range voltage source with variable input voltage, then *α* will have a value between zero and one. Furthermore, *α* will get closer to zero as the operating voltage ranges of the converter become wider. Therefore, *α* can be interpreted as a measure of the converter’s wide range operation, the wider the operating voltage the smaller *α* becomes.

Consequently, as *α* becomes smaller the converter design becomes harder, because demanding wider voltage ranges from the converter will effectively reduce the number of admissible converter designs. It will be shown later that this will impose more restrictive constraints on the parameters of the converter which in turn means that the allowed region in the parameter space will be smaller.

From the definition of α and the realistic values of the input and output voltages, the range of α is restricted to be: $\:0<{\upalpha\:}<1$. This interval can be derived from the facts that $\:{\upalpha\:}\:=\:1$ can only happen if the corresponding input and output voltages are equal, while $\:{\upalpha\:}\:=\:0$ can only happen if the minimum or the maximum of input voltage is zero.

#### The maximum normalized frequency

To express the maximum normalized frequency *f*_*n, max*_ in terms of *f*_*n, min*_ and *λ*, solving (25) for *f*_*n, max*_,

26 $$\:{f}_{n,max}=\sqrt{\frac{{M}_{min}\lambda\:}{-1+{M}_{min}+{M}_{min}\lambda\:}}$$

To rewrite (26) to be in terms of *f*_*n, min*_ and *λ* only, substitute (18) into (24) then into (26).

To obtain positive real value for *f*_*n, max*_ from (26), the radicand must be positive real. After some algebraic manipulations, the following validity conditions are derived,

27 $$\:(0<{M}_{min}\le\:1\:\&\:\lambda\:>\frac{1-{M}_{min}}{{M}_{min}})\:\:OR\:\:(1<{M}_{min}<\sqrt{2}\:\&\:\lambda\:>0)$$

The conditions in (27) are additional constraints that must be satisfied, however they are not satisfied automatically if (16, 17) are satisfied. Therefore, these additional necessary conditions will now be analyzed in detail.

There are two ways to satisfy (27) either satisfy the first condition or the second condition. The first condition in (27) states that there is an additional constraint on *M*_*min*_ and *λ* when *M*_*min*_ is less than 1. From the voltage gain equation, it can be deduced that the gain is less than 1 only when the normalized frequency is higher than 1. Rearranging the first condition,

28 $$\:{M}_{min}>\frac{1}{1+\lambda\:}$$

**Fig. 7:**
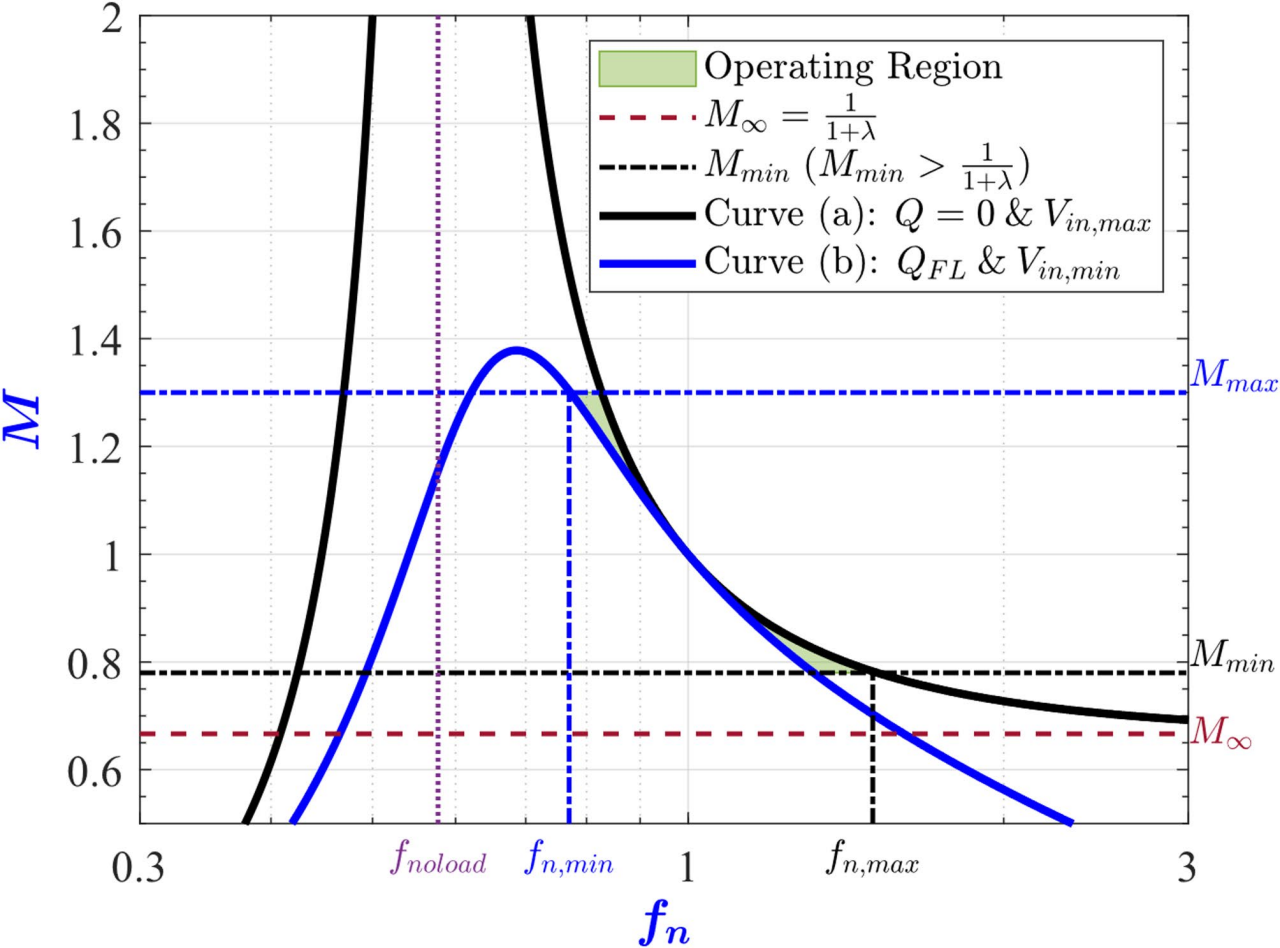
Semi-Log graph for the lower bound on the minimum voltage gain M_min_, M_∞_ is the lowest possible voltage gain when Q = 0 and f_n_ > 1.

The inequality (28) is recognized as the well know expression for maximum voltage gain available when the *f*_*n*_ > 1^24^. This can be seen clearly in Fig. 7.

The first condition in (27) can be interpreted as follows, after *f*_*n, min*_ and *λ* are chosen in the allowed region in Fig. 6, *M*_*min*_ is calculated from (24), and if *M*_*min*_ < 1 then condition (28) must be checked. If condition (28) is respected by the chosen *f*_*n, min*_ and *λ* then we can proceed with the next step in the design. Otherwise, the choice is invalid and another combination of *f*_*n, min*_ and *λ* must be chosen. This process must be repeated until a valid combination is reached, later we will show another procedure which does not require any repetitions. The first condition can be summarized as follows, if *M*_*min*_ < 1 then condition (28) will restrict the allowed region in the parameter space in Fig. 6. Consequently, condition (28) can be regarded as an indirect constraint on *f*_*n, min*_ and *λ*.

The second condition in (27) states that if *M*_*min*_ is larger than 1 then there are no additional constraints imposed. Collectively (27), states that if the minimum voltage gain *M*_*min*_ (point D in Fig. 3) is higher than 1 then the entire allowed region in Fig. 6 is indeed allowed. However, if *M*_*min*_ is less than or equal to 1 then there is an additional constraint which will effectively reduce the allowed region in Fig. 6.

**Fig. 8:**
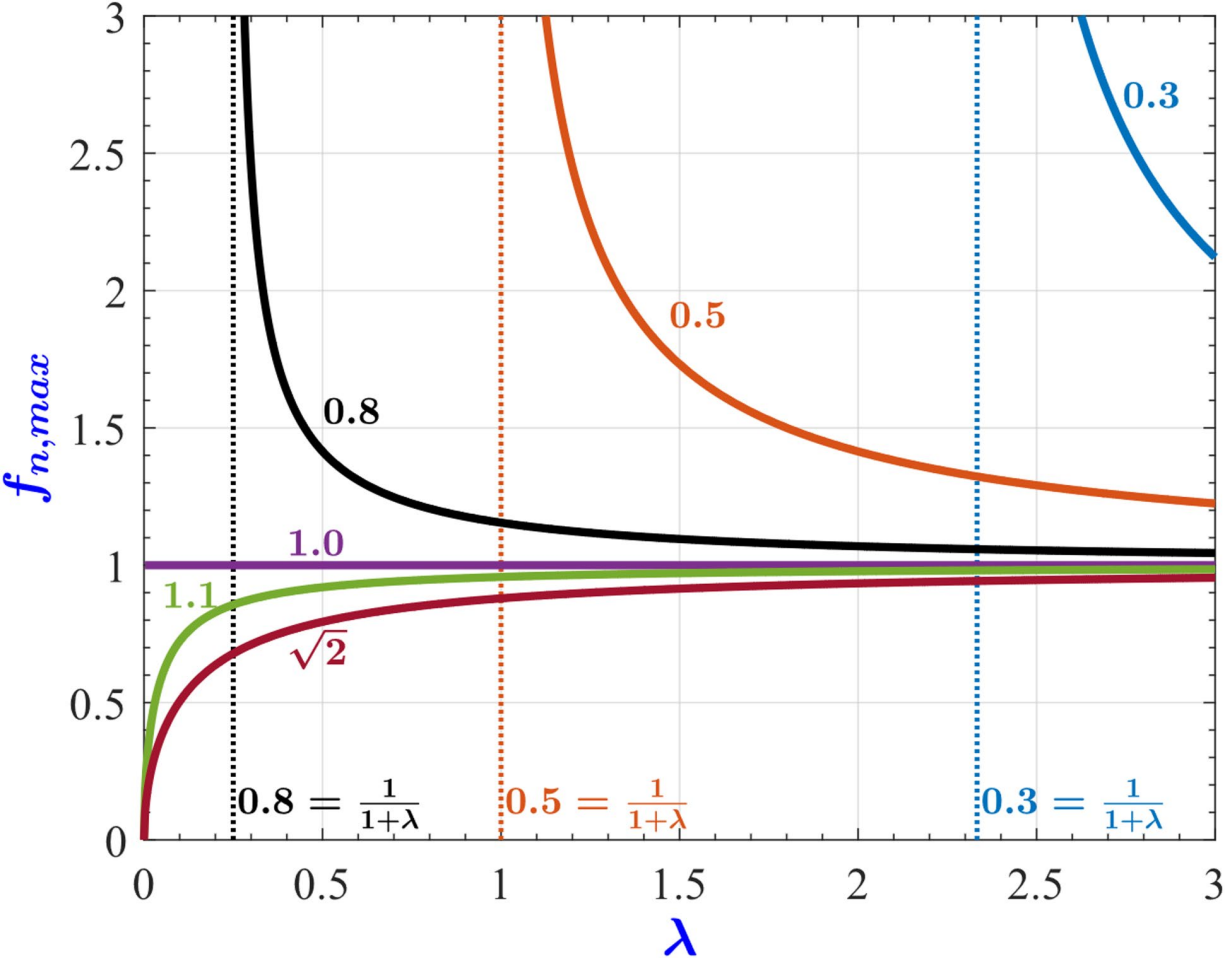
Plot of f_n, max_ in terms of λ at different values of M_min_.

To get a better understanding of (26 and 27), Eq. (26) is plotted in Fig. 8, where the asymptotes are defined by the equation $\:\lambda\:=\frac{1-{M}_{min}}{{M}_{min}}$. Hence, for the cases where *M*_*min*_ < 1, as λ approaches $\:\frac{1-{M}_{min}}{{M}_{min}}$ the *f*_*n, max*_ approaches infinity. While for the cases where *M*_*min*_ > 1, *λ* can take any value and *f*_*n, max*_ will always be between 0 and 1.

**Fig. 9:**
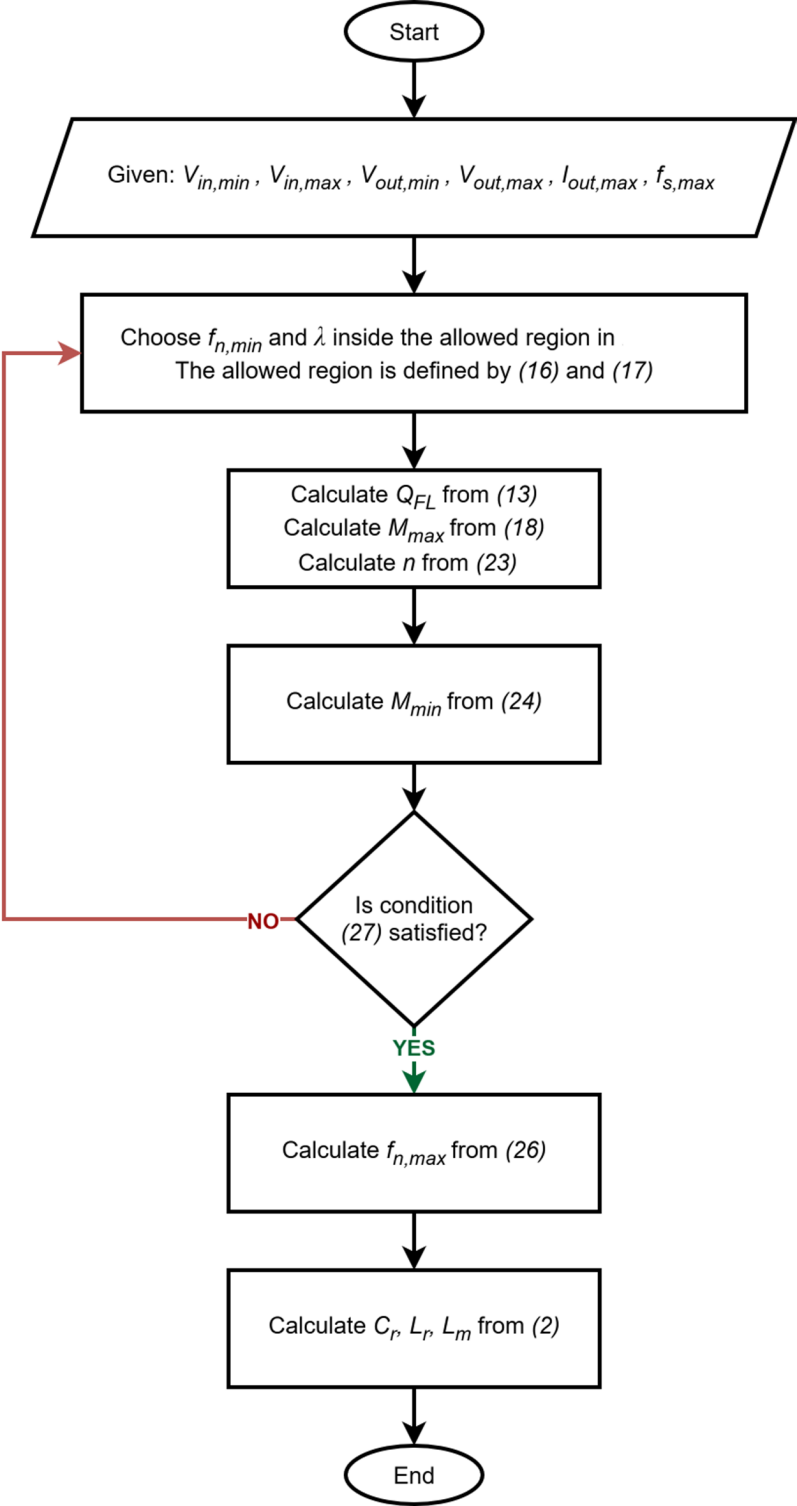
Flowchart for the initial design procedure.

In the previous sections, expressions for each of the converter’s parameters in terms of *f*_*n, min*_ and *λ* have been derived. The design procedure is summarized in the flowchart of Fig. 9. Clearly, this flowchart is iterative, since choosing any combination of *f*_*n, min*_ and *λ* inside the allowed region in Fig. 6 does not always guarantee that the condition (27) is satisfied. To overcome this limitation, in the next section Fig. 6 will be updated in such a way as to incorporate the condition (27) into the allowed region of *f*_*n, min*_ and *λ*, to guarantee that the chosen *f*_*n, min*_ and *λ* will always satisfy the condition (27). Later, a new flowchart will be presented for the updated design procedure.

#### Incorporating the additional constraints into the allowed parameter space

The first step is to rewrite the conditions (27) in terms of *f*_*n, min*_ and *λ*. Substituting (18) into (24) then into (27), then simplifying considering (16, 17),

$$\:IF\:(\:\begin{array}{cc}0<{M}_{min}\le\:1\:\:\&\:0<\alpha\:\le\:\frac{1}{\sqrt{2}}\:)&\:\end{array}$$

29 $$\:\frac{{f}_{n,min}^{2}(-1-{f}_{n,min}^{2}(-1+{\alpha\:}^{2})+\sqrt{-1+2{\alpha\:}^{2}+{f}_{n,min}^{2}(-2+{f}_{n,min}^{2})(-1+{\alpha\:}^{2})})}{-2+4{f}_{n,min}^{2}+{f}_{n,min}^{4}(-2+{\alpha\:}^{2})}<\lambda\:\le\:\frac{{f}_{n,min}^{2}}{2-2{f}_{n,min}^{2}}$$

$$\:IF\:(\:\begin{array}{cc}0<{M}_{min}\le\:1\:\:\&\:\frac{1}{\sqrt{2}}<\alpha\:<1\:)&\:\end{array}$$

30 $$\:\frac{{f}_{n,min}^{2}(-1-{f}_{n,min}^{2}(-1+{\alpha\:}^{2})+\sqrt{-1+2{\alpha\:}^{2}+{f}_{n,min}^{2}(-2+{f}_{n,min}^{2})(-1+{\alpha\:}^{2})})}{-2+4{f}_{n,min}^{2}+{f}_{n,min}^{4}(-2+{\alpha\:}^{2})}<\lambda\:\le\:\frac{{f}_{n,min}^{2}(-1+\sqrt{-1+2{\alpha\:}^{2}})}{2(-1+{f}_{n,min}^{2})}$$

$$\:IF\:(\:\begin{array}{cc}1<{M}_{min}<\sqrt{2}\:\:\&\:\frac{1}{\sqrt{2}}<\alpha\:<1\:)&\:\end{array}$$

31 $$\:\frac{{f}_{n,min}^{2}(-1+\sqrt{-1+2{\alpha\:}^{2}})}{2(-1+{f}_{n,min}^{2})}<\lambda\:\le\:\frac{{f}_{n,min}^{2}}{2-2{f}_{n,min}^{2}}$$

Conditions (29), (30) and (31) are equivalent to (27). These conditions can be summarized as follows:

If $\:\alpha\:\le\:\frac{1}{\sqrt{2}}$ then $\:{M}_{min}\le\:1$ and this will restrict the allowed space in Fig. 6 (allowed *f*_*n, min*_ and *λ*) as per the condition (29). If $\:\alpha\:>\frac{1}{\sqrt{2}}$ then either $\:{M}_{min}\le\:1$ or $\:{M}_{min}>1$ depending on *λ* and *f*_*n, min*_, if $\:\lambda\:\le\:\frac{{f}_{n,min}^{2}(-1+\sqrt{-1+2{\alpha\:}^{2}})}{2(-1+{f}_{n,min}^{2})}$ then $\:{M}_{min}\le\:1$ and the condition (30) applies otherwise $\:{M}_{min}>1$ and the condition (31) applies.

Since *M*_*min*_ is not known at the beginning of the design process, it is crucial to rewrite the antecedent conditions inside the “If ” parenthesis in the conditional expressions (29), (30) and (31) to be in terms of *λ* and *f*_*n, min*_ instead. Accordingly, (29), (30) and (31) can be rewritten as:

- $$\:IF\begin{array}{cc}(0<\alpha\:\le\:\frac{1}{\sqrt{2}}\:)&\:Then,\:\end{array}\:\:\:0<{M}_{min}\le\:1$$ and the condition (29) is applicable.
- $$\:IF\begin{array}{cc}(\frac{1}{\sqrt{2}}<\alpha\:<1\:\&\:\:\lambda\:\le\:\frac{{f}_{n,min}^{2}\left(-1+\sqrt{-1+2{\alpha\:}^{2}}\right)}{2\left(-1+{f}_{n,min}^{2}\right)}\:)&\:Then,\end{array}\:\:\:0<{M}_{min}\le\:1$$ and the condition (30) is applicable.
- $$\:IF\begin{array}{cc} \begin{array}{cc}(\frac{1}{\sqrt{2}}<\alpha\:<1\:\&\:\lambda\:>\frac{{f}_{n,min}^{2}(-1+\sqrt{-1+2{\alpha\:}^{2}})}{2(-1+{f}_{n,min}^{2})}\:)&\:Then,\:\end{array}&\:1<{M}_{min}<\sqrt{2}\end{array}$$ and the condition (31) is applicable.

**Fig. 10:**
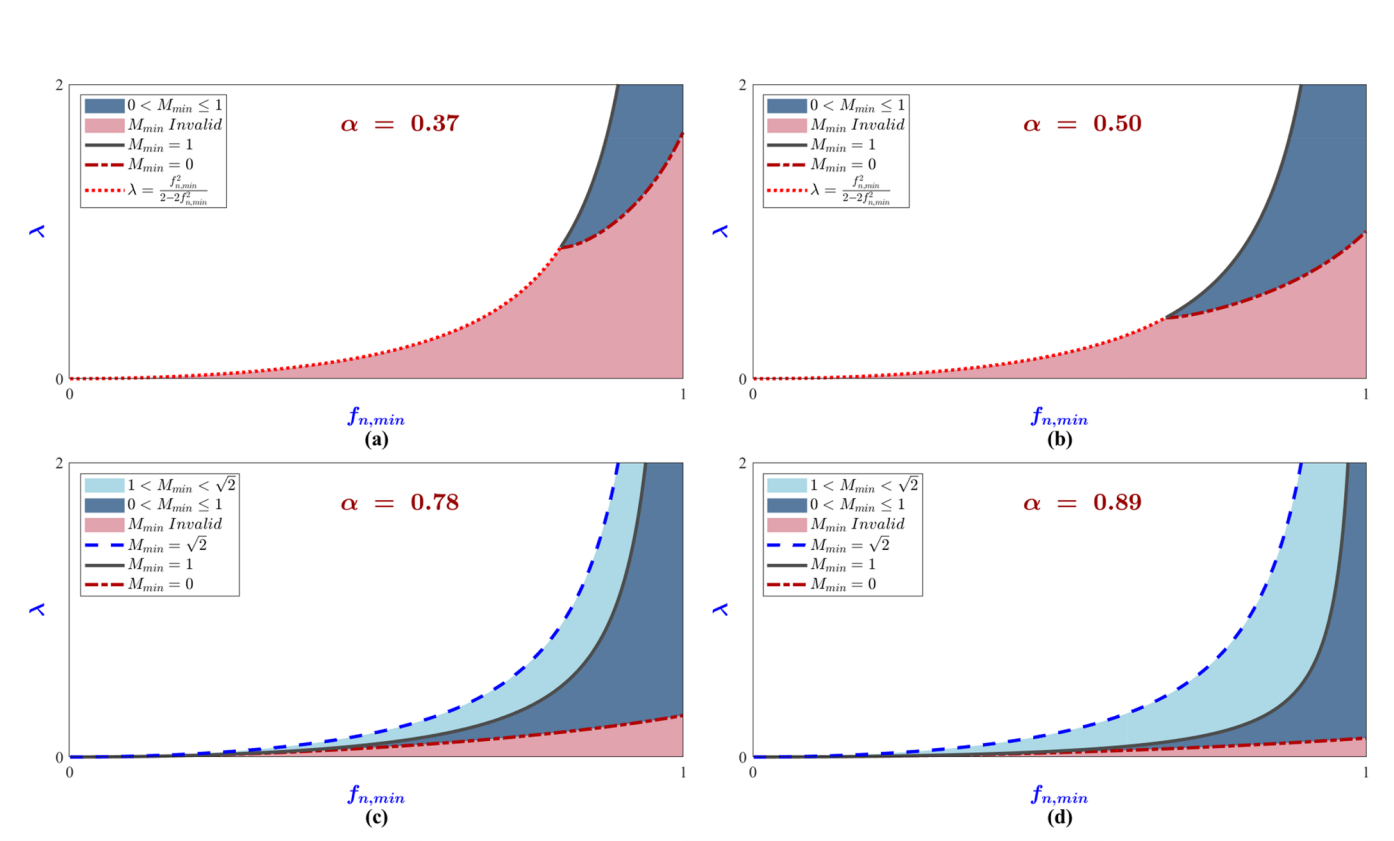
Visualizing conditions (29), (30) and (31) at different values of α. The pink region is the original allowed space in Fig. 6 which would result in an invalid value for M_min_ (does not satisfy (27), the dark blue region is where 0 < M_min_ ≤ 1 (conditions (29) and (30)), the light blue region is where 1 < M_min_ < √2 (condition (31)). (**a**) α = 0.37, (**b**) α = 0.5, (**c**) α = 0.78, (**d**) α = 0.89.

In summary, as *α* decreases, which indicates that wider operating voltage range is required, the smaller the allowed region in Fig. 6 becomes since more restriction are imposed on the allowed *f*_*n, min*_ and *λ* combinations. In Fig. 10, conditions (29), (30) and (31) are plotted for different values of *α* to visualize how *f*_*n, min*_ and *λ* allowed space in Fig. 6 changes as *α* changes.

As *α* starts very small in Fig. 10a, condition (29) severely restricts the allowed space, the value of the minimum voltage gain *M*_*min*_ can never exceed one. As α increases in Fig. 10b, the restriction imposed by the condition (29) is relaxed, thus enlarging the allowing space, while *M*_*min*_ is still restricted to not exceed one. In Fig. 10c, α is increased above the value of $\:1/\sqrt{2}$, the allowed space is further enlarged. Moreover, the allowed space now consists of two regions with different properties, one region is where $\:0<{M}_{min}\le\:1$ as per the condition (30) while the other region is where $\:1<{M}_{min}<\sqrt{2}$ as per the condition (31). In Fig. 10d, α is increased even further, the allowed space is further enlarged while maintaining the two distinct regions that first appeared in Fig. 10c.

#### Design equations for the LLC circuit elements

From the equations for *λ* (16), *Q*_*FL*_ (13), *f*_*n, max*_ (26), and (2), the LLC circuit parameters can be calculated

32 $$\:{L}_{r}=\frac{4{f}_{n,max}{n}^{2}{Q}_{FL}{R}_{L}}{{f}_{s,max}{\pi\:}^{3}},\:\:{c}_{r}=\frac{{f}_{n,max}\pi\:}{16{f}_{s,max}{n}^{2}{Q}_{FL}{R}_{L}},\:\:{L}_{m}=\frac{4{f}_{n,max}{n}^{2}{Q}_{FL}{R}_{L}}{{f}_{s,max}{\pi\:}^{3}\lambda\:}$$

The proposed algorithm will optimize the efficiency of the converter in such a way that the peak efficiency will occur at a load resistance near the value of *R*_*L*_. Therefore, it is common to choose this resistance value to be the most common load of the converter during operation^15^ which usually is the full-load condition, thereby *R*_*L*_ can be calculated from $\:{R}_{L}=\:{V}_{out,max}/{I}_{out,max}$^10^.

## The proposed updated design procedure

**Fig. 11:**
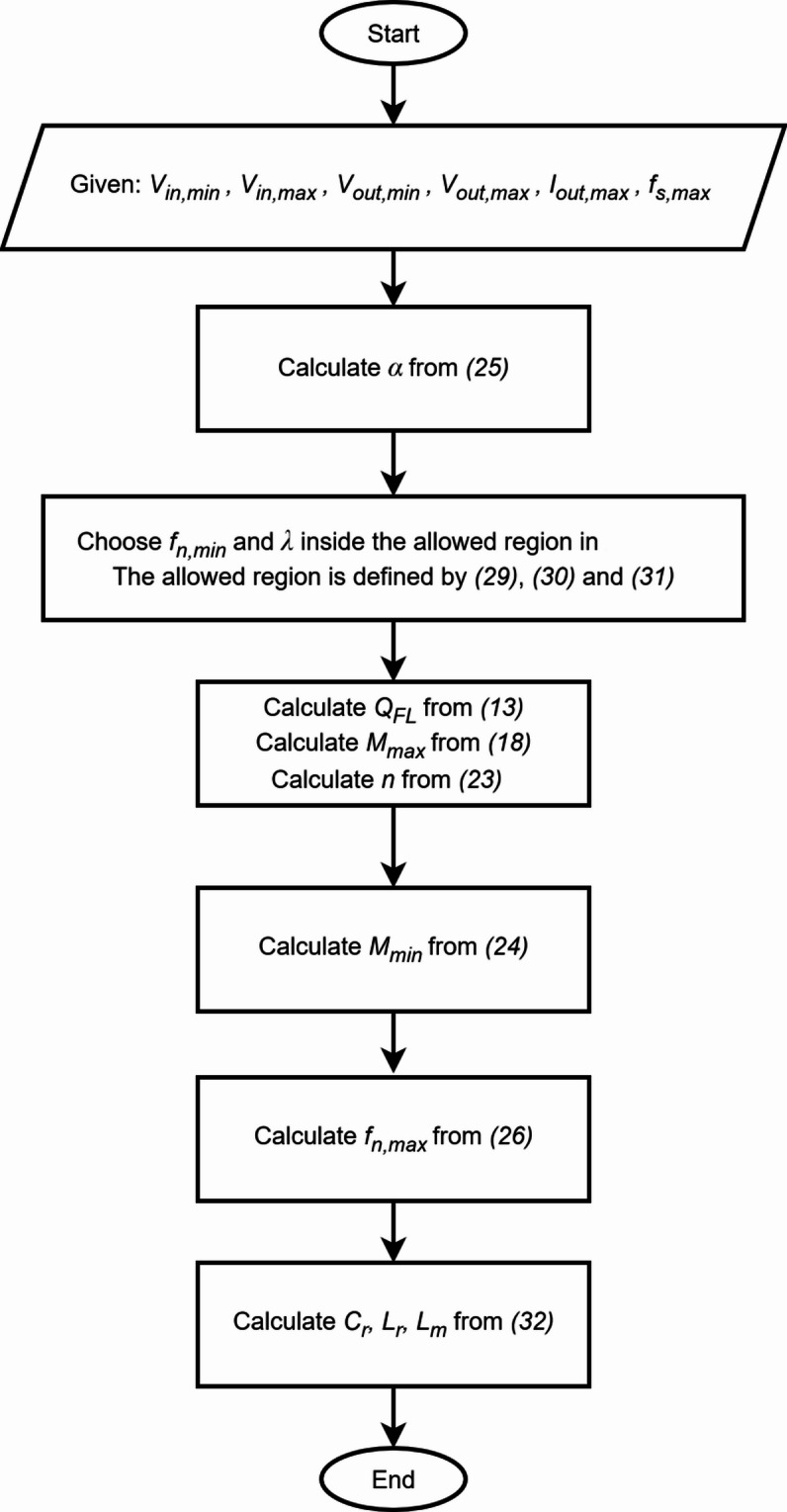
Flowchart for the updated design procedure.

In this section the updated design procedure is summarized in the flowchart of Fig. 11 and then applied to design the LLC converter. As can be seen in the flowchart, there are no loops or conditions to check, the design procedure is straightforward. Any combination of *f*_*n, min*_ and *λ* that satisfy the conditions (29), (30) and (31) are guaranteed to produce a valid design. As long as $\:0<\alpha<1$ is satisfied, the proposed algorithm will always generate a valid optimal design, however, if the input or output ranges are very wide then the generated design could be impractical for implementation.

The converter designed in this section shall meet the following specifications: the minimum input voltage *V*_*in, min*_ = 320 V, the maximum input voltage *V*_*in, max*_ = 370 V, the maximum switching frequency *f*_*s, max*_ = 315 kHz, the maximum load current *I*_*out, max*_ = 3.0 A, the minimum output voltage *V*_*out, min*_ = 35 V, the maximum output voltage *V*_*out, max*_ = 165 V, the full-load Resistance *R*_*L*_ = 165 V / 3.0 A = 55 Ω. Following the design procedure flowchart Fig. 11,

Step 0: Update the maximum output voltage *V*_*out, max*_ to incorporate a 10% safety factor. *V*_*out, max*_ = 181.5 V.

Step 1: Calculate *α*, from (25), *α* = 0.166778.

**Fig. 12:**
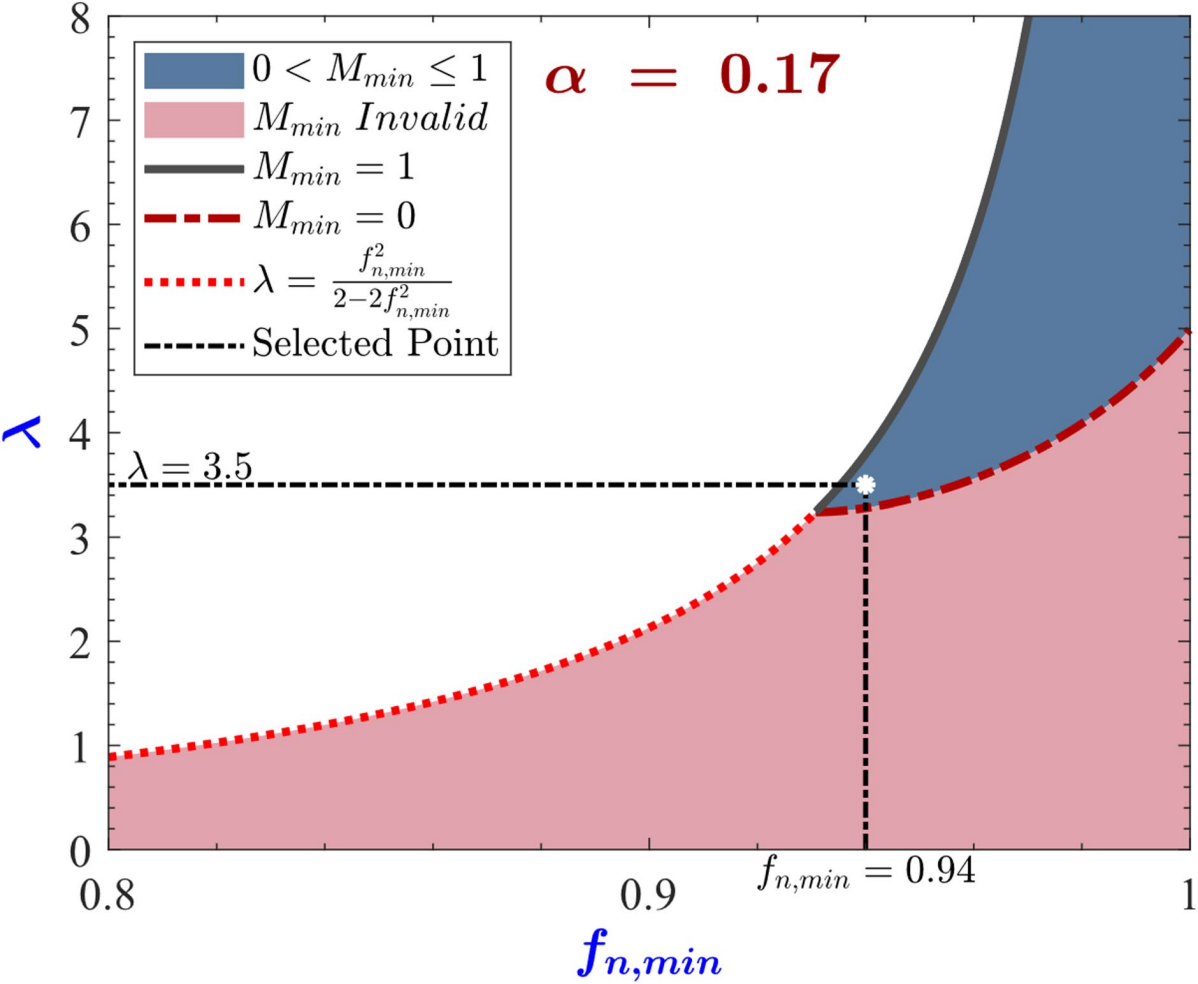
f_n, min_ and λ parameter space (Design Area) for the designed converter.

Step 2: To plot the *f*_*n, min*_ and *λ* allowed space, it must be determined which of the conditions (29), (30) and (31) is applicable. Considering the value of *α* calculated in step 1, (29) is the applicable condition. The *f*_*n, min*_ and *λ* parameter space is plotted in Fig. 12.

Step 3: Choose *f*_*n, min*_ and *λ* within the allowed space in Fig. 12. the entire region $\:0<{M}_{min}\le\:1$ in Fig. 12 is valid in the sense that any point chosen there would lead to a converter that can achieve all of the specifications. As can be seen in Fig. 12, the combination (*f*_*n, min*_ = 0.94 and *λ* = 3.5) is chosen within the allowed space.

Step 4: Calculate the converter parameters, *Q*_*FL*_ = 3.72 from (13), *M*_*max*_ = 1.41 from (18), *n* = 1.243 from (23), *M*_*min*_ = 0.235 from (24), *f*_*n, max*_ = 3.76 from (26).

Step 5: Calculate the converter LLC values, *L*_*r*_ = 487.4 µH, *C*_*r*_ = 7.4 nF, *L*_*m*_ = 139.2 µH from (32).

It is clearly evident that the proposed design procedure is straight forward and does not require any iterations.

It is preferable to design the converter with small frequency variation range and compact components. From (32), it can be shown that increasing *f*_*s, max*_ and decreasing *f*_*n, max*_ will decrease the size of the components. Moreover, based on Fig. 8, Eqs. (26) and (27), to decrease *f*_*n, max*_, *λ* must be increased to be much higher than $\:\frac{1-{M}_{min}}{{M}_{min}}$. However, based on (2), increasing λ will increase the size of *L*_*r*_. Therefore, there is a tradeoff between decreasing the size of the components (*L*_*r*_, *L*_*m*_, and *C*_*r*_) and decreasing the frequency variation range ($\:{f}_{n,max}-\:{f}_{n,min}$). Nevertheless, using the closed form equations derived in this paper, the designer can control the design tradeoffs while ensuring that the design will meet the specifications.

Smaller values of *f*_*n, max*_ are usually preferred to reduce the frequency variation range. In that case, the proposed methodology gives quite accurate results. However, for large values of *f*_*n, max*_, the accuracy of this procedure is reduced due to the influence of higher-order harmonics that are neglected in the FHA approach, which is an inherent limitation for all design procedures based on the FHA. Nevertheless, the design procedure remains valid and gives acceptable results. Therefore, in applications where large values of *f*_*n, max*_ is unavoidable, it is recommended to validate this analytical approach with simulations.

Smaller values of *λ* are usually preferred to allow the physical integration of the *L*_*r*_ and *L*_*m*_ into a single transformer. However, the value of *λ* in this case study is large, thus it is not possible to physically integrate the *L*_*r*_ and *L*_*m*_. This limitation is a direct consequence of requiring the converter to operate with wide voltage range in both the input (320–370 V) and output (35–165 V). In other words, the wider the voltage ranges, the higher *λ* becomes. This is a known limitation of wide voltage range design techniques, for example^10^.

## Simulation results

To validate the proposed design strategy, presented in Sect. “The proposed updated design procedure”, the designed LLC converter has been simulated at a steady state under different scenarios using LTSPICE. The parameters and specifications of the designed LLC resonant converter have been tabulated in Table 2.

**Table 2:** Key parameters of the designed Converter.

Parameter	Symbol	Value	Loss model used in simulation
Input Voltage	*V*_*in, min*_ – *V*_*in, max*_	320–370 V	–
Output Voltage	*V*_*out, min*_ – *V*_*out, max*_	35–165 V	–
Output Current	*I*_*out, max*_	3 A (at full load)	–
Output Power	*P*_*out, max*_	495 W (*V*_*out, max*_ x *I*_*out, max*_)	–
Power MOSFETs	M₁, M₂	2×STP12NM50	Shichman-Hodges model (SPICE VDMOS Level 1 model) With R_ds, on_ = 300mΩ at I_DS_= 5.8 A
Power diodes	D₁ – D₄	4×STTH802	Shockley diode model with parasitic (SPICE D model) With V_total drop_=922mV at I_D_=5.2 A
Inductance-ratio	*λ*	3.5	–
Transformer turns ratio	*n*	1.243	–
Resonant capacitance	*Cr*	7.4 nF	Assume constant ESR = 50 mΩ
Magnetizing inductance	*Lm*	139.2 µH	Assume constant ESR = 150 mΩ
Resonant inductance	*Lr*	487.4 µH	Assume constant ESR = 200 mΩ
Dead-time	*ΔT*	350 ns	–

**Fig. 13:**
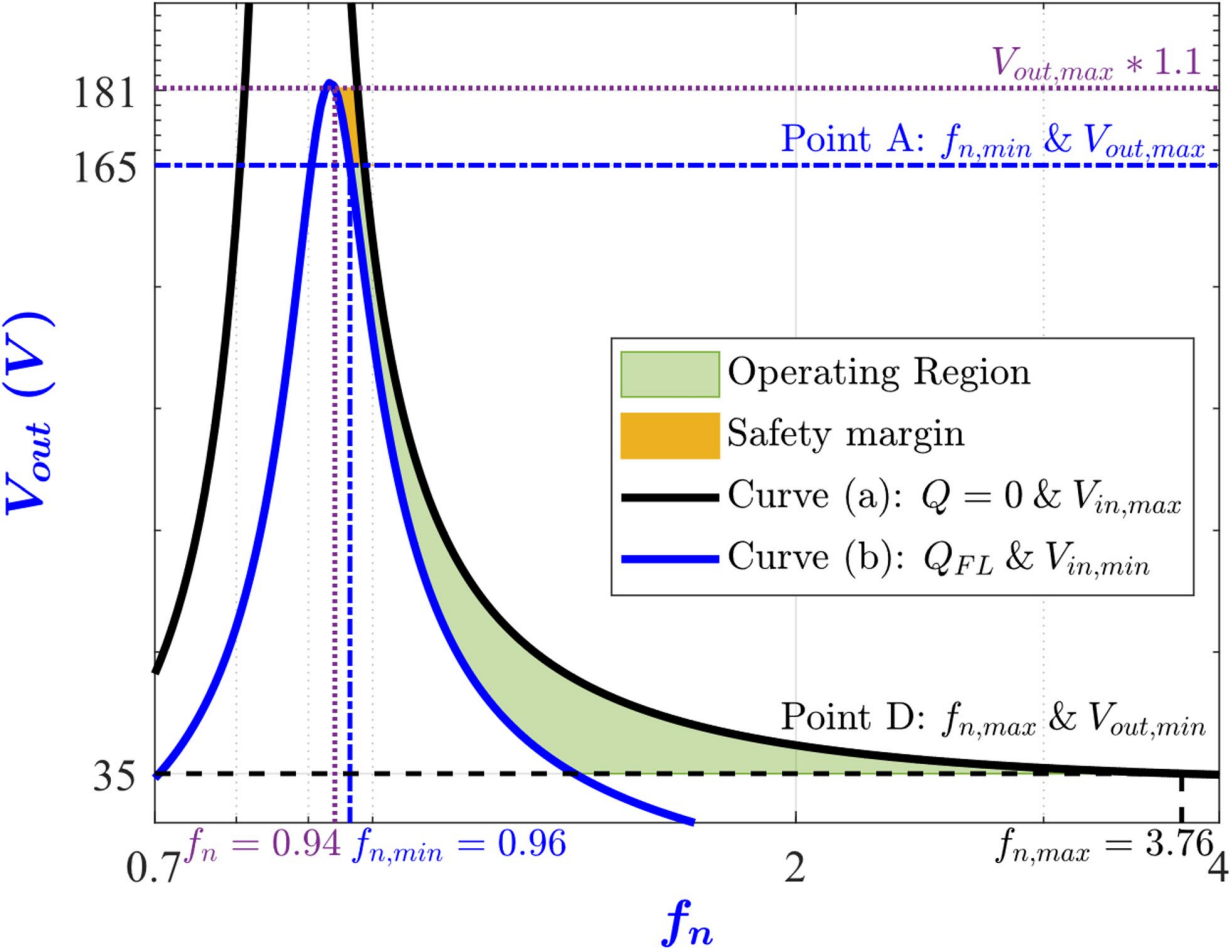
Semi-log graph (f_n_ in log scale) of the theoretical dc output voltage V_out_ versus the normalized switching frequency f_n_ of the designed converter.

Figure 13 shows the output voltage transfer function of the designed converter versus the normalized switching frequency under the two worst-case scenarios. The first scenario is operating at the full-load condition with minimum input voltage, curve (b) in Fig. 13. This scenario represents point A in Fig. 3 when operating at *f*_*n, min*_. The peak voltage is equal to 181.5 V, achieved at *f*_*n*_ = 0.94, which is 10% higher than the output voltage required of 165 V, achieved at *f*_*n, min*_ = 0.96. The second scenario is operating under the no-load condition with maximum input voltage, curve (a) in Fig. 13, which represents point D in Fig. 3, where the output voltage is 35 V at *f*_*n, max*_ = 3.76. This demonstrates that the converter succeeded in achieving the full operating voltage ranges as per specifications.

**Fig. 14:**
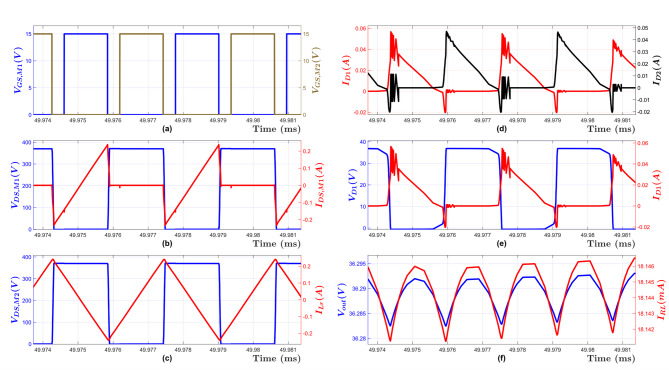
Simulation Waveforms at No load and V_in, max_ = 370 V, (**a**) Mosfet Gate voltages for M1 V_GS, M1_ and M2 V_GS, M2_, (**b**) MOSFET M1 drain-source voltage V_DS, M1_ and drain-source current I_DS, M1_, (**c**) Resonant Tank input voltage V_DS, M2_ and input current I_Lr_, (**d**) Rectifier current for Diode D1 I_D1_ and Diode D2 I_D2_, (**e**) Diode D1 voltage V_D1_ and current I_D1_, (**f**) Load Voltage V_out_ and current I_RL_.

Figure 14 shows the converter primary-side and secondary-side waveforms when maximum input voltage *V*_*in, max*_ (370 V) is applied to the converter under the no-load condition. The output voltage is successfully regulated at the minimum value *V*_*out, min*_ (35 V) through employing the maximum switching frequency *f*_*n, max*_.

**Fig. 15:**
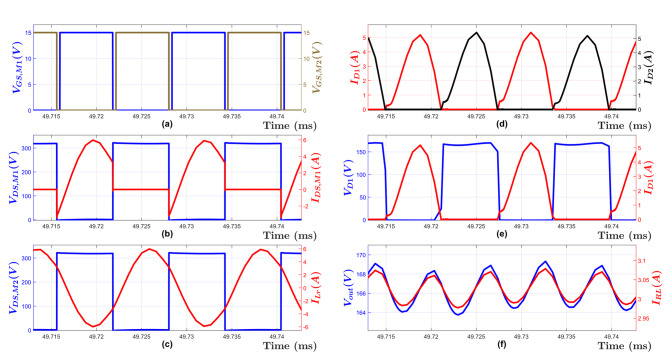
Simulation Waveforms at Full Load and V_in, min_ = 320 V, (**a**) Mosfet Gate voltages for M1 V_GS, M1_ and M2 V_GS, M2_, (*b*) MOSFET M1 drain-source voltage V_DS, M1_ and drain-source current I_DS, M1_, (**c**) Resonant Tank input voltage V_DS, M2_ and input current I_Lr_, (**d**) Rectifier current for Diode D1 I_D1_ and Diode D2 I_D2_, (**e**) Diode D1 voltage V_D1_ and current I_D1_, (**f**) Load Voltage V_out_ and current I_RL_.

Figure 15 illustrates the converter primary-side and secondary-side waveforms to deliver maximum power to the output load (Full-load condition) when minimum input voltage *V*_*in, min*_ (320 V) is applied to the converter. As shown in this figure, the maximum output voltage *V*_*out, max*_ (165 V) is achieved through employing the minimum switching frequency *f*_*n, min*_.

**Fig. 16:**
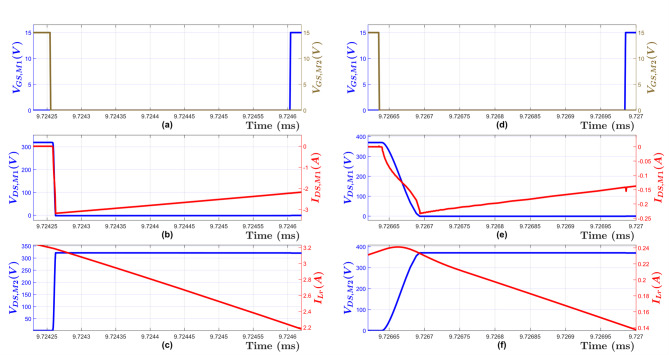
(LEFT) Simulation Waveforms at Full Load and V_in, min_ = 320 V. (**a**) MOSFET Gate voltages for M1 V_GS, M1_ and M2 V_GS, M2_, (**b**) MOSFET M1 drain-source voltage V_DS, M1_ and drain-source current I_DS, M1_, (**c**) Resonant Tank input voltage V_DS, M2_ and input current I_Lr_, (RIGHT) Simulation Waveforms at No load and V_in, max_ = 370 V. (**d**) MOSFET Gate voltages for M1 V_GS, M1_ and M2 V_GS, M2_, (**e**) MOSFET M1 drain-source voltage V_DS, M1_ and drain-source current I_DS, M1_, (**f**) Resonant Tank input voltage V_DS, M2_ and input current I_Lr_.

To demonstrate the ZVS operation for the designed LLC converter, Fig. 16 shows the high side MOSFET (M1) drain to source voltage being reduced to zero before the gate voltage is applied. Hence, the ZVS operation is achieved even under the two worst-case scenarios. As shown in this figure, the dead-time must be chosen to be more than the time required for the MOSFET drain to source voltage to be reduced to zero, otherwise the ZVS property will be lost^9,10,19^. However, increasing the dead-time will result in lower efficiency, thus a design tradeoff. In this case, a deadtime of 350 ns was deemed adequate.

**Fig. 17:**
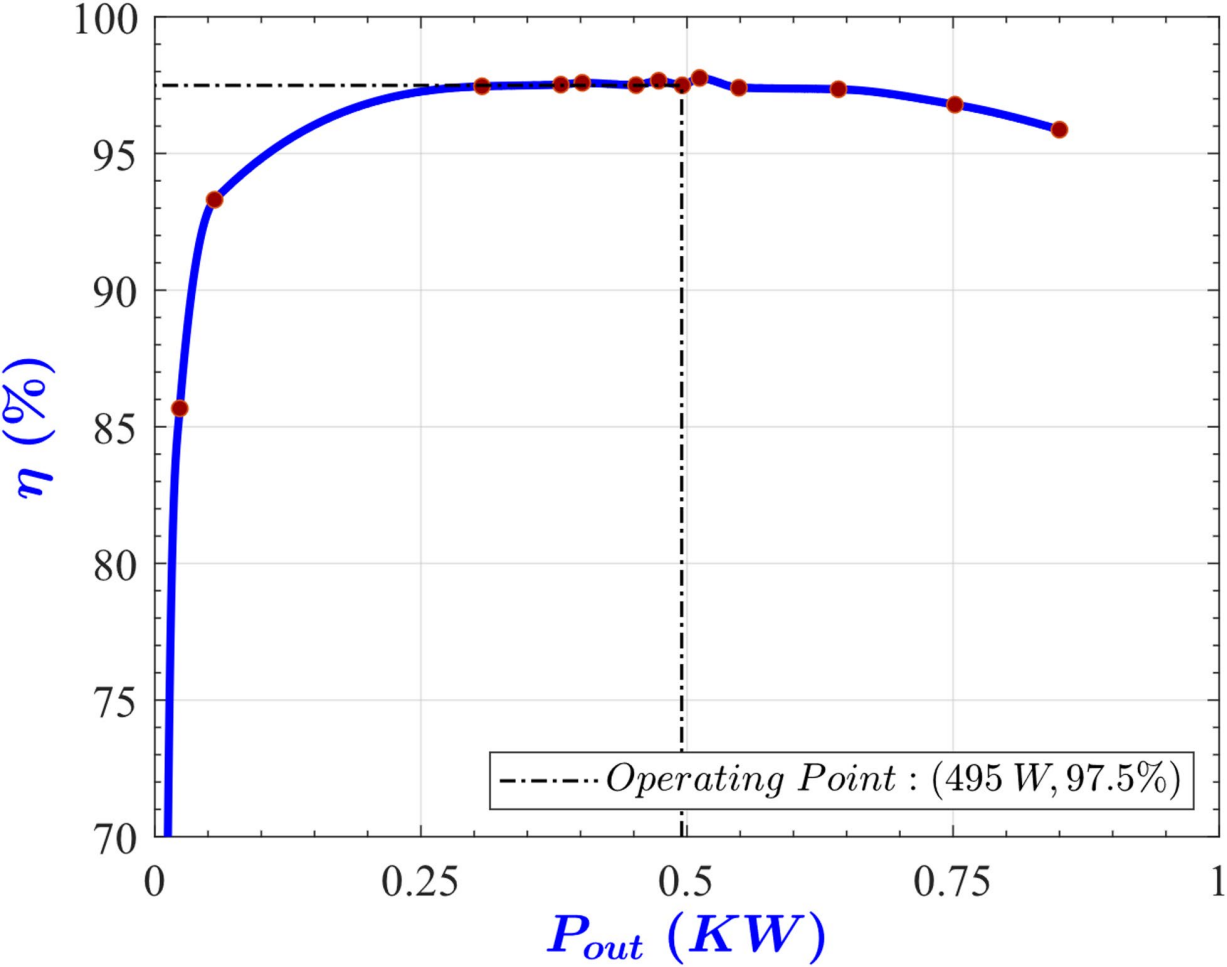
Efficiency η versus output power P_out_ at V_in, max_.

Figure 17 illustrates the converter efficiency versus its output power at *V*_*in, min*_ and *V*_*out, max*_ while the load resistance is changed in steps. According to this figure, at the full load, 495 W, an efficiency of 97.49% is achieved, while the absolute maximum efficiency is 97.76% achieved at an output power of 511 W. This means that the efficiency at the full load condition is less than the maximum efficiency by around 0.3%, this deviation can be explained as a consequence of the FHA method ignoring the harmonics and the underlying assumptions used in deriving (6). This result demonstrates that the designed converter successfully attained maximum efficiency near the full-load operating point.

To validate the accuracy of the simulation results, a comparative study is summarized in Table 3 comparing the proposed and other published methods. For the comparison to be unbiased, the converters must be designed for the same operating conditions based on the same circuit topology and simulated using the same model. Thus, for each published method, a converter is designed using the proposed approach to operate in the same conditions with the same maximum switching frequency, then both are simulated at full load (minimum input voltage, maximum output voltage and maximum output current at the minimum switching frequency). Compared to the other methods, the proposed approach tends to improve efficiency while using lower switching frequency at full-load. However, this comes at the expense of higher *L*_*r*_*/L*_*m*_ ratio and larger switching frequency range. The lower switching frequency at full-load is a direct consequence of choosing a small value for *f*_*n, min*_ in Fig. 12. Accordingly, the switching frequency range can be reduced by choosing a higher value for *f*_*n, min*_ .

**Table 3:** Comparative study using the same simulation model.

Method	Vout (V)	Vin (V)	Pout (W)	Lr (uH)	Lm (uH)	Cr (nF)	*N*	F_s_ (KHz)	Efficiency (%)
^10^	35–165	320–370	495.13	243	161	6.6	2.33	106–315	96.58
Proposed	35–165	320–370	495.18	487.4	139.2	7.4	1.24	81–315	97.49
^26^	250–300	300	352.41	2.86	500	621	0.5	100–170	97.89
Proposed	250–300	300	353.32	28.07	140.03	116	0.52	80–170	98.01

## Conclusion

In this paper, a novel optimal design procedure for improving the efficiency of the LLC resonant converter at a specific load while operating over a wide output voltage range has been presented. Moreover, all possible circuit configurations that satisfy the wide output voltage range constraint, the ZVS condition for the whole operation, and the max efficiency at a predetermined output load have been identified. This approach simplifies the design of the converter to merely selecting the values of two independent parameters to be within some permissible parameter space and the rest of the converter parameters can be derived accordingly. Analytical expressions for the converter design parameters have been derived in closed form, which ensures that the converter design optimization can be done without the need of the slow and computationally expensive numerical optimization algorithms. Additionally, the design methodologies that rely on numerical optimization algorithms can benefit from the proposed method in selecting the initial circuit designs to be used as a starting point for these algorithms. Thus, ensuring that the initial designs are both feasible and near the optimal design, thereby reducing the time needed to reach the global optimal design of the LLC resonant converter.

## Data Availability

The datasets used and/or analyzed during the current study available from the corresponding author on reasonable request.
